# Emotional journey of wives of spouses diagnosed with bipolar I disorder: moving from vicissitude towards reconciliation

**DOI:** 10.1080/17482631.2021.1946926

**Published:** 2021-07-02

**Authors:** Tehreem Fatima Naqvi, Rabia Dasti, Nasar Khan

**Affiliations:** a Centre for Clinical Psychology, University of the Punjab, Lahore, Pakistan; b Centre for Clinical Psychology, University of the Punjab 54590 Quaid-e-Azam Campus, Lahore, Pakistan; c Division of Developmental Disabilities, Queens University, Kingston, Canada

**Keywords:** Lived experience, wives, bipolar disorder and emotional journey

## Abstract

**Purpose:**

Our present study was a qualitative investigation intending to explore the emotional journey of wives whose spouse has been diagnosed with Bipolar I Disorder, using a phenomenological design.

**Method:**

Semi-structured face to face interviews were conducted with 5 wives of already diagnosed Bipolar I Disorder patients to uncover their lived experience in terms of the emotional journey they had had. For data analysis, we used Hycner’s explicitation process. Moreover, for data verification we employed the strategies of frequent debriefing sessions peer review and member checks.

**Results:**

Our analysis revealed six major themes encapsulating the participants emotional journey. These included *Shock, Betrayal and the Incomprehensible, Apprehensions and Uncertainty, Anger and Irritability, Loneliness and Helplessness, Compassion and Acceptance* and *Reconciliation.*

**Conclusion:**

It became clear to us that wives of individuals diagnosed with Bipolar I Disorder are on a continuous emotional journey dealing with the burden, stress, complications, uncertainty and making many sacrifices along the way. Our study highlighted many culture specific factors of the phenomenon. This insightful exploration has opened up new horizons to conceptualize the challenges of wives dealing with an ailing spouse in the context of a Pakistani society.

Bipolar Disorder is a mental illness that is prevalent in 1 to 3.7% of the population and it is believed that by the year 2020, it will become the sixth leading reason of disability worldwide amongst all the medical illnesses (Miklowitz et al., 2007; Murray & Lopez, 1996; Reinares et al., 2006). The onset of Bipolar Disorder usually takes place in late adolescence or early adulthood. Fifty percent of the cases start before age 25 (Kessler et al., 2005). Even when on regular pharmacological treatment, the illness has a high recurrent rate with at least 40–60% of patients go through a minimum of one relapse of mania or depression within two years (Gitlin et al., 1995). Numerous impairments in school, occupation and social functioning are seen in patients even in a remission phase (Ball et al., 2006; Blairy et al., 2004; Dion et al., 1988; Michalak et al., 2004). The disorder comes with substantial effects on the social functioning of the patient and their relatives, affecting the quality of their lives (Rouget & Aubry, 2007). Compared to healthcare providers and family members, the spouse of a bipolar patient is the most vulnerable among all the caregivers as they are believed to have the maximum investment in their relationship (Pruchno & Resch, 1989). Bipolar Disorder tends to erode the quality of almost every interpersonal relationship of the patient, and marriage seems to be no exception.

Being and staying in a relationship with someone diagnosed with Bipolar Disorder is a great challenge. The conflict in these marriages is much higher than in others. Some authors have called bipolar marriages as “intermittently incompatible”, ones i.e., while they can stay stable otherwise, whenever the patient gets ill, the marriage is threatened by massive upheaval (Greene et al., 1976). Spouses carry with them persistent emotional pressure every day, building an emotional vacuum in which they find themselves continuously trapped. They tend to go through immense aloneness nearing on dissociation that surrounds them, especially when the spouse is unwell (Lawn & McMahon, 2014). For many spouses the emotional impact comes in the face of helplessness, anxiety, loneliness, frustration, hypervigilance, resentment and fear of relapses and the future ahead (Granek et al., 2016). This emotional vacuum travels and surfaces for the spouse as anger, grief, frustration etc. The illness blocks the sharing of their fears, needs and hopes with their spouse increasing on the aloneness and isolation in their relationship (Lawn & McMahon, 2014).

The lack in attention given to spouse caregivers comes as a surprise as among all the discharged patients hospitalized for psychiatric disabilities, 35–40% of them go back to live with their partners. Furthermore, several spouses are also raising children at a higher risk of having a mental illness, such as Bipolar Disorder (Corring & Pateman, 2003). This volatility in the relationship leads to many contemplating on and many a times even getting divorced (Granek et al., 2016). Separation and divorce is higher in couples having a spouse diagnosed with Bipolar Disorder in comparison to the general population (Brodie & Left, 1971; Kessler et al., 1998). However, in the Pakistani society, getting divorced or even separated is not an easy option to go with especially for a woman. The concept of divorce still remains a taboo and is not an acceptable option by most of the society no matter the problems in the marriage. Women are expected to stay in stressful conditions, compromise and not think about divorce (Shamsi, 2018). Thus, most of the women even when faces with tremendous difficulties in a marriage be it incompatibility with spouse, abuse or the spouse being mentally or physically ill, ends up staying stuck in marriages. Having children, no financial stability, unsupportive families and society at large etc. serve as reasons to staying in the marriage (Zahid, 2018) Therefore, the aim of my research was to explore the emotional journey of wives living with spouse diagnosed with Bipolar I Disorder within a Pakistani cultural context.

## Method

This phenomenological study utilized a qualitative research methodology to explore the emotional journey the wives whose spouse have been diagnosed with Bipolar I Disorder have lived through.

## Rationale of the study

A marital relationship can be a critical health resource for adults. Being married typically is expected to lead to greater availability of emotional social support, meaning and purpose in life, and social control (Umberson, 1987). However, any number of things, from work stress to money issues, can lead to arguments and put strain on a marriage. But when one partner has bipolar disorder, simple stressors can reach epic proportions. The patient themselves face emotions including shame, sadness and self-doubt (Granek et al., 2016). Side by side, the disorder poses way more challenges for the spouse. In case of patients with affective disorders the spouses experiences severe burden due to the chronicity, episodic nature of the illness and the resulting uncertainty created by intermittent dysfunctionality experienced as a result of being symptomatic, whether it’s a manic or depressive episode (Borowiecka-Kluza et al., 2013; Chakrabarti et al., 1992; Perlick et al., 1999). Although there is an abundance of literature that addresses issues associated with family members of persons with severe mental illness, the literature concerning the effect of bipolar disorder on their spouses is extremely limited (Lam et al., 2005). Instead of focusing exclusively on the reduction of patient’s illness and improvement in their interpersonal functioning, attention could profitably be directed to the experiences of spouses. Also the available literature have all been carried out in the West where moving out of a marriage or a relationship is comparatively easier and acceptable. On the other hand, in the Eastern cultures, despite going through several problems in one’s marriage, the pressures from family and society and the stigma that divorce carries makes it more challenging for individuals to move out of a marriage. Therefore, due to the different marital relationship dynamics as shaped by the Pakistani culture, there is a need to deeply explore the emotional journey of spouses of Bipolar I Disorder patients in order to capture the richness of the issues posed by a chronic and debilitating condition of bipolar disorder and the challenges this condition imply on the institution of marriage, which forms the basic social unit in the Pakistani society. Thus, the present study aimed to understand the emotional journey of wives whose husband has been diagnosed with Bipolar I Disorder using a qualitative approach.

## Research question

The objective of this study was to gain an increased understanding of how does living with a husband having Bipolar Disorder emotionally impacts the spouse. The answer for the following question was explored from the perspective of the wife.

What is the perception and experience of living with a spouse diagnosed with Bipolar I Disorder?

This research question was followed by sub-questions such as “How did you get to know about your spouses’ illness?”, “How did you feel when you got to know about it?”, “How do you feel about it now?”, “How do you express your feelings to your spouse?”, “Has the illness influenced the expression of your feelings to your spouse? If yes, in what way?” “How do you see your emotional relationship with your spouse over the period of your marriage?”, “Do you want to tell anything else related to your emotional journey so far?”

## Sampling strategy

For this study, we employed criterion sampling as the criteria for this study was a participant’s experience of living with a spouse diagnosed with Bipolar I Disorder. Criterion sampling works best when all participants studied are representative of individuals who have experienced the phenomenon (Creswell, 2007).

### Inclusion criteria

Participants who were living with a spouse diagnosed with Bipolar I Disorder were included. The couples should be married for at least 4 years and have at least 1 child. The patient must have been diagnosed with having Bipolar I Disorder prior to getting married. All patients will be receiving some kind of treatment (either medical or therapy). The participant had to be able to understand the letter of information and be willing to provide consent. The participant had to be willing to participate in at least two semi-structured interviews discussing her marriage and family. 5 such wives were included in the study.

### Exclusion criteria

It was the diagnosis of a major mental illness for spouses. Spouses who have reached a formal diagnosis of a mental disorder before marriage were also excluded. If this was the second marriage for either of the spouse, it led to the exclusion of that participant from the study. Those who were separated, divorced or whose spouse has died were also excluded.

## Data collection

Our study underwent an ethics approval by the Independent Departmental Doctoral Program Committee of our department that is responsible for evaluating the ethics and methodology of any research being carried there. After receiving ethical and methodological approval for the current research we, developed two paper instruments for the purposes of this study, a socio-demographic questionnaire and a semi-structured interview protocol. For the development of the interview protocol, the previous literature and theories on the current topic were consulted. They were used as the driving force in the formulation of the interview protocol. This was then followed by the scrutiny of the interview protocol by 5 professionals (3 working clinical psychologists and 2 experienced academicians in the field). The suggestions and recommendations given by them were duly noted and incorporated. Then permission was sought from the heads of all hospitals, from which the sample was to be obtained. The mental health professionals in all the hospitals were approached and requested to give reference of any individual with Bipolar I Disorder, meeting the inclusion/exclusion criteria. Reminders were repeatedly given to the respected professionals through personal visits and telephone calls. Countless visits were made to OPD’s of the selected hospitals. Side by side, we also distributed flyers explaining our research and the criteria of our sample to our contacts including colleagues, peers, students, relatives etc. so that they could refer relevant participants for our research in case they know of any. These flyers were also posted on different social media bipolar support groups. We ended up recruiting, 5 participants who consented and also met the study criteria and were then interviewed for the study. Amongst them, 2 were recruited from the OPD of a local hospital, 2 participants were referred by our colleagues while the last one came forward after coming across our study flyer on a social media group.

Prior to the interview, an informed consent form was duly signed. All interviews were conducted using a semi-structured interview guide by a single interviewer. Patients’ spouse was interviewed independently to ensure privacy and the ability to talk easily and candidly about their experiences. Initially, study participants were inquired about their history (upbringing, interests, education, etc.) not only for research purposes but also as a rapport building to ease into interview gradually. As we proceeded with the interview, follow-up questions were asked in order to gather more details with respect to their experiences. Interviews were audiotaped with a digital recorder. The time duration of all the interviews ranged from approximately 60–90 minutes. All participants were contacted a second time for some additional information. All the interviews were carried out in Urdu language as all the participants were fluent at that.

Each recorded interview was then transcribed, removing all identifiable information from the transcripts. In addition to the use of semi-structured interviews, we also penned down our personal reflections after each interview and incorporated them in the analysis. All the field notes were taken, describing the thoughts, feelings or assertions we had before, during and/or after any interview session. These notes helped us to add context to my transcribed data and they also reflected our vigilance and alertness during the interview. All the data was sorted in a file that included the informed consent, the socio-demographic questionnaire, the verified transcripts and my personal reflections for each participant. It was then secured in a locked filling cabinet with no identifying data included in it. Each file was maintained under codenames only to ensure confidentiality.

## Participant characteristics

The participants’ (wives of spouses diagnosed with Bipolar I Disorder) age ranged between 24–31 years old. Two of them had completed their intermediate degree, two had a graduation degree while the last one had done MBA. Apart from one participant who resided in a nuclear family setup, the rest lived in a joint family system. 3 participants came from a lower middle-class setup while the other 2 from a moderately middle class ones. The marriage of all the participants was an arranged one. Participants were married for a minimum of 4 years and a maximum of 7 years and had at least 1 child from the marriage. None of the participants have ever been married before nor did anyone suffered from any mental disorder.

## Explicitation of the data

We analysed the data by employing the steps outlined by Hycner (Groenewald, 2004). This explicitation process includes five “steps” or phases as follows

1. Bracketing and phenomenological reduction.
2. Delineating units of meaning.
3. Clustering of units of meaning to form themes.
4. Summarizing each interview, validating it and where necessary modifying it.
5. Extracting general and unique themes from all the interviews and making a composite summary.

In the first step, based on Hycner’s recommendation, we listened to the recording of every interview, in order to become acquainted with the words of each participant and transcribed them. This step involved getting closer to the original data from the participants. We read the transcribed interviews repeatedly and took initial notes and developed a rough coding procedure for each interview. Next, we derived units of meanings for each participant by extracting all the significant statements that explained the lived experience of the participant. Each and every statement is of great value in phenomenology, so we treated each statement as having equal value and then read and re-read the data to ensure that no significant statement is missed out and we listed down all these statements separately and eliminated redundant and repetitive statements. After rigorous examination of the units of meanings, themes were generated by combining the units of meanings and a summary that incorporated all the themes from the data was developed. This summary was validated from the participant and modified if required. After, the process of extracting themes from each participant’s data was done, We tabulated a collective chart to outline themes from all the interviews, including the unique themes that we came across. If substantial differences were present, clustering of common themes was carefully avoided. These themes were finalized after getting three peer reviews (feedback discussed ahead) from practicing clinical psychologists, with a clinical experience of at least 5 years with 2 of them having experience of conducting qualitative enquiry as well. The explicitation process was concluding in the result section which reflects the context from which the themes emerged.

## Data verification

The credibility of qualitative research is frequently questioned, perhaps because the concepts of reliability and validity are not catered in the same way as in a quantitative research. Therefore, strategies such as frequent debriefing sessions. Peer review and member checks (Shenton, 2004) were employed.

## Results

Five wives living with a spouse diagnosed with Bipolar I Disorder were interviewed for this study. After analysing the data, the following themes emerged from this research study: Shock, Betrayal and the Incomprehensible, Apprehensions and Uncertainty, Anger and Irritability, Loneliness and Helplessness, Compassion and Acceptance and Reconciliation. The themes served as a reminder of the emotional impact on study participants’ while living with a spouse diagnosed with Bipolar I Disorder. Participants often discussed an array of emotions that they have experienced since they first got to know about their spouses’ illness. The journey of each participant seemed like a roller coaster ride of emotions going up and down the tracks and impacting each participant emotionally in their own way.

## Shock, betrayal and the incomprehensible

The participant’s marital life took a dramatic turn the first time their spouse became ill. They were not prepared for their spouse’s significant behavioural changes as none of them previously knew about the illness. When asked about how they felt when they first got to know about the illness, most of the participants reported feeling shocked when they first saw their spouse experiencing a manic/depressive episode. Many seemed unable to comprehend what is happening while many felt betrayed at the hands of their in-laws or their husband. They did not understand what was happening, or what to do.

Participant 1:

“I got really upset because all of this was very sudden and I had no such thought in my mind.

He had all this even before marriage and I got to know about it afterwards. When I got to know I used to cry a lot, thinking that someone should have taken us into confidence. And I love my husband a lot, and he also really loves me so I kept thinking that at least he should have shared this with me.”

Participant 2:

“I was really shocked, I was unable to understand what was going on … .I was in great shock and used to stay really sad.”

Participant 3:

“I felt betrayed, betrayal it is isn’t it? So betrayal it was … we also had a fight over it, I fought a lot … I really felt like someone had deceived me big time. I felt really bad then, I got really sad and my mom too.”

## Apprehensions and uncertainty

As the initial shock and incomprehensibility subsided, it was soon taken over by apprehensions and uncertainty for many participants. Dwelling about the reoccurrence of the illness, the likelihood of personality changes in the spouse and the possibility that it could run in future generations troubled left some participant apprehensive, worried and uncertain about the future. Participants went on to share how they had numerous worries and questions about their spouse’s illness and how it made them anxious and worried about the uncertainty that came with it.

Participant 1:

“Initially I got really anxious thinking that if this illness is recurring, it can occur again at any time. This was a really big worry. And secondly I was worried that now even after getting better will he be like before or not … so I was really worried that what will happen now.”

Participant 4:

“I was also told that this is not an issue that has happened once, it can happen anytime in life. This was the reason I got really worried because you have to spend your whole life with someone, and you know that they can get ill anytime, so you do get worried.”

## Anger and irritability

Anger and irritability were prevalent emotions that many participants experienced especially during the active phase of their spouse’s illness. Many shared getting angered easily at every other thing and feeling irritated all the time. For some the anger stemmed from the blaming by their spouse in a manic episode and others around for absolutely anything going wrong. While the anger and irritability also resulted from spouse’s total refusal to cooperate with them in responsibilities that had to be taken care of. One participant also shared how she sometimes displaced the emotions on her little daughter when unable to get her emotions under control.

Participant 1:

“I also often got angry … his situation was like this that it felt like handling a stubborn child … I got irritated myself in all these circumstances I couldn’t do anything properly … I would get really angry, but I would obviously keep it in … I would also argue a lot during those days.”

Participant 5:

“I was really angry during this time it was like I would explode at anything even if it was a minute little thing.

I felt very angry … I got really angry that fine it is an illness like I try from my side to understand but you know that there is a point to which I can understand.

I am human too.”

## Loneliness and helplessness

Loneliness and helplessness came out to be a significant emotional impact that almost all the participants went through. They shared how after the onset of their spouse’s illness and once they got over the initial shock, the feeling of being alone and helpless in this situation eventually took over. The rapid change that occurs in the spouse’s behaviour during the active phase of the illness, leads them feeling lonely. Many also shared how their spouses completely change as a person making it impossible to talk to them. Thus, the feeling of loneliness takes over and saddens them. Some participants also went on to report how they now feel hopeless about their spouse’s illness and have no choice but to accept it. Everything seems completely out of their hands with no control that they could exercise.

Participant 1:

“I feel really alone, I have to keep my problems to myself. I wish all this shouldn’t have been like this and I could share everything like before, but it is really difficult now.”

Participant 3:

“Sometimes I feel like this is not in my control. When a person feels completely helpless, that is how I feel sometimes … I feel completely alone. I feel like it was my bad luck that he turned out to be sick. Like even when with him I feel there is no one, I am completely alone.”

Participant 4:

“So, whatever it is I’m handling it alone and not telling anyone … I feel more alone in these circumstances. I had never imagined my life to be like the one I’m experiencing now.”

## Compassion and acceptance

Feelings of compassion towards her spouse seemed to have now overcome a participant’s life. Dealing with the spouse compassionately and with patience was the way for her to make her peace with the situation and attain some emotional stability. She shared how she focuses on the positive sides of all this and tries to recall how supportive her husband is when he is not ill. For some participants acceptance was the dominant feeling that had settled in now. They sorted to making a compromise with their relationship and just accepting how things are. While explaining her spouse’s illness, one participant even alluded it as a *“sotan”* (second wife) to her. However, they had chosen to accept that this is how life is going to be and made their peace with it.

Participant 1:

“I try to handle him with patience … I was thinking more about the positive side of handling it and thank God I got through it. It passed. I understand him as he is very supportive when he’s not ill.”

Participant 3:

“It feels like we have made a compromise … this illness is my *sotan* (second wife of husband).”

Participant 4:

“Slowly slowly I started to adjust with these things … sometimes his condition gets worse, but I have adjusted with this.”

## Reconciliation

The many roles and responsibilities that the participants had to play and look over accompanied with an unsupportive, unaccommodating, insulting or abusive spouse got a bit too much for them many a times. The irritation and helplessness they would experience due the chronicity of the illness and the repetitive cycle of relapse nudged them to making the decision of just leaving their husband. All the participants talked about times they had given up and decided to leave their spouse and what ultimately made them stay. Having children came out as a reoccurring reason that most of the participants gave for their stay, along with thinking about their spouse’s well-being and having no one else to go to.

Participant 2:

“I have thought many times but as I told you all doors are closed behind me … it had come in my mind that how will I live with him, I should go somewhere, do something for myself but now I have children so it’s really difficult.”

Participant 3:

“My mother-law made a big issue that if you don’t want to stay then we will keep your daughter … even if I leave then where will I go, I only have one daughter … I have also aged, who will marry me now … .I also think about him … my sister-in-laws will make my life hell, however and whatever I am, at least I have a roof over my head.”

Participant 5:

“I have only one son because of whom I think we are together. Because if he was not there then maybe I would have started a business or something with my sister and even if I would not have gone to my parents home, I would have at least gotten separated.”

## Discussion

The overall aim of this study was to explore the lived experiences of wives whose spouse have been diagnosed with Bipolar I Disorder and to understand their emotional journey. The analysis revealed overriding dominant themes exfoliating the emotional journey of wives whose spouse have been diagnosed with Bipolar I Disorder.

Mental illnesses can walk-in with an unimagined cruelty, slashing through a family’s home like an unexpected tornado that gives no time for preparation. It comes quickly and leaves behind destruction and confusion as its repercussion. The experience of the wives living with a spouse diagnosed with Bipolar I Disorder started from the first time they experienced a manic/depressive episode of their spouse before their own eyes. While the experience was unique and distinct for each participant, they shared a mutual feeling of being shocked, betrayed and unable to fathom what was going on as all of them were unaware of their spouse’s illness before marriage. A spouse who had previously been loving, caring, understanding and supportive, suddenly became an angry, irritated, abusive and distant person who was unrecognizable now. This was followed by numerous subsequent emotions leading to some form of emotional reconciliation with the situation. Living with Bipolar Disorder can be like riding an emotional roller-coaster and it came out just like that for my study participants too as evident in their responses in the study. Karp’s (2002) stages of the emotional ride in a caregiving experience talks about the first stage of *Emotional anomie* that comes before a firm and formal diagnosis. The sudden changes in the behaviour of a mentally ill individual are almost always incomprehensible. The people around are left feeling anxious, fearful, and above all, completely confused. They are left bewildered about the chaos around and uncertain as to how to feel about it. While the second stage of *Getting a Diagnosis* brings hope and compassion for many caregivers, in the case of a mental disorder, other more negative feelings start to sneak into a caregiver’s mind. They expect the ill person to reciprocate their feelings of sympathy and be more responsive and understanding. Feeling of anger and resentment soon takes over a caregiver upon *Perceiving Illness Permanency*. Caregivers surrender to the tough reality that the expectations and desires they had for the ill individual in their life will not be realized by them. Their increasing loneliness is another source for the caregiver’s frustration. They become isolated because of the increasing demands of managing the illness. The increased isolation and feelings of aloneness in spouse of a person with Bipolar Disorder creates a feeling of their lives being surreal (Lawn & McMahon, 2014). They go through a persistent emotional burden that they have to carry with them every day. Many caregivers feel lonely and isolated in process of caregiving and experience difficulty in maintaining a normal life (Jonsson et al., 2011). Tranvag and Kristoffersen (2008) in their study on the experience of by a spouse/cohabitant of a person with Bipolar Disorder also talked about the emotional upheaval of the participants. The spouses/cohabitants reported not understanding what was going on, or what they can do. Their life got chaotic, erratic and incomprehensible. They recounted how the terrifying and unfathomable life situation gave rise to uncertainty. Similar emotions were experienced by my research participants who shared how shocked and confused they were seeing their spouse in an active phase of the illness for the first time. They also went on to feeling anger and irritability which was followed by loneliness soon after. Apprehensions and uncertainty about the illness and the future was also observed. Some have now accepted and made a compromise while one participant had developed feelings of compassion and focused on the positive aspects of the situation. Iseselo et al. (2016) cited that caregivers learn to accept and reconcile with the disability or unexpected behaviour of the relative who is mentally ill in order to avoid the frustration and disappointment that could result from the patient’s deviant behaviour. Similarly, Granek et al. (2018) found that partners of bipolar patients particularly discussed that it was helpful to dodge strong emotional reactions, use acceptance and take it one day at a time during the more trying times. Other participants talked of not spending much of their time thinking about the effects of Bipolar Disorder excessively, and instead trying to focus on positive aspects. They deliberated over the significance of love and tolerance. Moving on to the fourth stage of (Karp, 2002) emotional ride in a caregiving experience, he described how the recognition by the family members of not being able to control the patient’s illness leads to the abandonment of their efforts to fix them and is shortly followed by acceptance. Acceptance usually comes in the form of the “4 C’s Mantra”:

“I did not cause it, I cannot control it, I cannot cure it. All I can do is cope with it”

The emotional ups and downs each participant went through were very much in line with the above-mentioned previous literature. This emotional instability and turmoil seemed to have been affecting the participant’s marital life in a detrimental way. Khalatbari et al. (2013) found out in their study on marital satisfaction and emotional stability that there is a direct and significant relationship between marital satisfaction and emotional stability. Individuals who experience emotional instability report lower levels of marital satisfaction. Emotional distress has also been associated with increase in marital conflicts (Conger et al., 1994) and hostile marital interactions (Skinner et al., 1992). Such emotional instability and distress resonated in some of the participants in the present research also, which reflects how these feelings have an immense impact on them and resultantly on the relationship with their spouse. While most of my study participants had either made a compromise and accepted their spouses’ illness and resultantly talked about the damaging effect of it on their marital relationship. Contrary to this, one of the participants talked about how she developed feelings of compassion towards her spouse and actively tries to focus of the positive aspects which in turn help her stay positive and enjoy a better relationship with her spouse. Bipolar Disorder can be a struggle to live with for couples and the individual. Having a deep understanding of how to feel about the situation is beneficial for sustaining a stable long-term relationship for couples with a bipolar spouse. To be able to enjoy the utmost quality of individual and shared life one can, focus on positive and productive efforts, rather than getting stuck on negative and damaging cycles (Brenner, 2018).

Bipolar Disorder with its uncertain and unpredictable nature brings a whole bunch of challenges for the people around especially the spouse. It has been seen that the volatile and inconsistent nature of the disorder sometimes causes the spouse to seriously contemplate over separation or in some cases even divorce (Granek et al., 2016). Separation and divorce figures of bipolar patients seem to be higher than in comparison to the population as a whole (Brodie & Left, 1971; Kessler et al., 1998). Many spouses also report to have never married or have children had they known about their spouses’ illness’ genetic inheritability (Targum et al., 1981). Keeping in mind all this, the present study included participants who were not only married, had children but also still staying in their marriage. It was interestingly observed in the theme above that all the participants had contemplated on the idea of separation from their spouse not once but quite a few times. Some had also gone back to their homes but returned soon after, the reason being lack of acceptance and support back home. Shamsi (2018) discussed how the women in the Pakistani society are made to believe that marriage is a one-way street with no way out. Even though facing several complications in one’s marriage, the pressures from family and society and the stigma that word divorce comes with makes it nearly impossible for women to move out of a failing marriage. Hearing statements like “Respectable women do not leave their husbands and their homes”, “*Hum izzat wallay log hayn* [We are respectable people]. Such people do not have divorced daughters” and “Where will you go? You have no one” are heard frequently in the Pakistani culture if and when a woman reaches out to the society be it her parents, siblings or any other means of help (Zahid, 2018). Thus, it seems easier for women to stay put in an unhappy marriage rather than move out and deal with the condescending society. The most common and the main reason for all of the participants to stay in marriage were their children. Participants shared how it might have been much easier and even possible to leave their spouse had it not been for their children. Sacrificing one’s own happiness for the happiness of your children with the hope of providing them a steady and secure environment to grow up in seems like the most rational thing to do (Shamsi, 2018). All these reasons force a woman to stay in a marriage against her will or many a times she is made to believe that that is actually her will. Having to accept and compromise with the feeling of being stuck in a relationship instead of being in a one with your own will and wish effects an individual’s quality of relationship in a negative way. This was evident in the verbatim of the participants as well when they talked about what made them accept the situation and eventually stay in the marriage wistfully; wishing things could have played out differently.

## Limitations and future suggestions

Due to numerous field and practical problems (such as time constraints) five wives living with spouse diagnosed with Bipolar I Disorder had been the only focus of our study. The study can be replicated by increasing the number of participants in another comparative study. Other types of research methodologies, research design and research methods can be utilized as well.

## Conclusion and implications

We explored the experiences, such as the emotional journey of wives living with a spouse diagnosed with Bipolar I Disorder. It became clear to us that such wives are on a continuous emotional journey dealing with the burden, stress, complications, uncertainty and making many sacrifices along the way. Going through an extremely challenging, demanding and for many, an abusive relationship the participants didn’t see a way out from it. Our study also highlighted many culture specific factors of the phenomenon. This insightful exploration has opened up new horizons to conceptualize the challenges of wives dealing with an ailing spouse in the context of a Pakistani society. The present study could be used as the basis for introducing family therapy in the future as an integral part of managing psychological illnesses like Bipolar Disorder focusing on emotional regulation for spouses assuming the role of caregivers. As seen in the results above, none of the participants knew about their spouse’s illness before marriage, thus, introduction of programs reducing stigma and labializations should be encouraged to promote transparent sharing of such information as early as possible.
